# Pyruvate Kinase, Inflammation and Periodontal Disease

**DOI:** 10.3390/pathogens10070784

**Published:** 2021-06-22

**Authors:** Melissa M. Grant

**Affiliations:** School of Dentistry, Institute of Clinical Sciences, University of Birmingham, 5 Mill Pool Way, Edgbaston, Birmingham B5 7EG, UK; m.m.grant@bham.ac.uk

**Keywords:** pyruvate kinase, glycolysis, inflammation, regulation

## Abstract

Pyruvate kinase (PK) is the final and rate-limiting enzyme in glycolysis. It has four isoforms PKM1, PKM2, PKL and PKR. PK can form homo tetramers, dimers or monomers. The tetrameric form has the most catalytic activity; however, the dimeric form has non-canonical functions that contribute to the inflammatory response, wound healing and cellular crosstalk. This brief review explores these functions and speculates on their role in periodontal disease.

Pyruvate kinase in the enzyme (EC 2.7.1.40) that catalyses the final step in glycolysis (Figure 1), the biochemical and metabolic pathway converting glucose to pyruvate, producing ATP and NADH. Pyruvate kinase is expressed as four isoforms in mammalian cells—PKM1, PKM2, PKL and PKR. PKL and PKR are produced by the liver and red blood cells, respectively, whilst PKM1 and PKM2 are alternative splice variants of the PKM gene. PKM1 is expressed in tissues requiring high catabolic activity, such as the heart, muscle and brain; PKM2 is expressed by proliferative cells, such as leukocytes.

The pyruvate kinase monomer is approx. 58 kDa in size and comprises four domains: A, B, C and N. Domains A and B bind the substrates (phosphoenolpyruvate (PEP) and ADP)) and metal ions (Mg^2+^ and K^+^) and catalyse the reaction to produce pyruvate and ATP. PKM1 constitutively organises as a tetramer, which is efficient at catalysis without an activator. On the other hand, PKM2 requires an allosteric regulator for high activity, forming monomers (80%), dimers (10%) or tetramers (10%) [1] in the absence of the allosteric regulator. Allosteric inhibitors, such as ATP, oxalate, alanine and phenylalanine, and post-translational modifications (reviewed by Prakasam et al. 2019 [2]), such as the nitrosylation of specific Cys [3], can promote the inactive dimeric and monomeric forms. The allosteric regulators bind in domain C, away from the catalytic site: the most well studied is fructose-1,6-bisphosphate (FBP). Five different mechanisms have been proposed for allosteric regulation (see Schormann et al. 2019 [1] for details). The possibility of modifying pyruvate kinase activity makes it a potential drug target: furthermore, although it is ubiquitous, there are differences between the control of the enzyme in different species, particularly between the kingdoms of life, making it an attractive though challenging target. The C domain also contains a nuclear localisation signal [4] sequence, and the N domain is very short (43 amino acids).

During inflammation, leukocytes are activated, and this causes a switch from a resting to a high metabolic state in these cells. This allows for the generation of large quantities of ATP and macromolecules required for the effector functions of immune cells: production of inflammatory mediators, rearrangement of the cytoskeleton and proliferation. PKM2 may have additional roles to that of mediating the production of pyruvate, however. In macrophages stimulated by lipopolysaccharide (LPS), PKM2 expression is induced, PKM2 translocates to the nucleus of the cells and forms a complex with transcription factor HIF1alpha, activating transcription of genes, such as IL-1beta [5], IL-6 [6] and HMGB1 [7]. Inhibition of PKM2 by shikonin or by knockdown of PKM2 expression prevents LPS driven release of these mediators [5]. In other situations, PKM2 has been shown to interact with tyrosine kinases, such as A-Raf, BCR-ABL and FGFR1 [8,9], RNA binding proteins, such as HuR and tristetraprolin, and even to Bcl-2, stabilising it during oxidative stress.

In neutrophils, changes in glycolytic phenotype were first observed in 1959 [10], and increases in the activity of PKM2 during activation by opsonized zymosan have been specifically demonstrated [11]. In cystic fibrosis, neutrophils display an increase in aerobic glycolysis in circulation as measured by the quantity of cytosolic PKM2 in these cells [12]. In response to PMA, neutrophils can form neutrophil extracellular traps (NETs): Aswathi et al. [13] explored the role of PKM2 in netosis and showed that there is a decrease in PKM2 activity but an increase in PKM2 dimerization, suggesting a non-canonical role for PKM2 in netosis. A further well-known role of neutrophils is degranulation; upon stimulation with FMLP, neutrophils degranulate and PKM2 is deposited into the extracellular space [14]. PKM2 in wounds is known to accelerate healing via the promotion of angiogenesis [14,15,16]. HIF1alpha is also active in neutrophils: the cells drive a hypoxic microenvironment through the great need for oxygen for energy and reactive oxygen species production. PKM2 dimers interact with and activate HIF1alpha, as mentioned before.

PKM2 has been found in overabundance in various inflammatory conditions, such as Crohn’s disease [17], hepatic disorders [18], renal disease [19] and Rheumatoid Arthritis [20], as well as in a wide range of cancers [21], including oral cancers, as measured in extracellular samples, such as serum and stool. In cancer, the overabundance of PKM2 is associated with a poorer outcome [22]. Several systematic reviews have explored the use of pyruvate kinase in the diagnosis of cancers: Hathurusinghe et al. [23] concluded that PMK2 was elevated in a range of gastrointestinal malignancies; Uppara et al. [24] demonstrated that PKM2 had relatively good sensitivity (79%) and specificity (80%) for screening for colorectal cancer, and Wang et al. [25] determined that PKM2 had moderate performance in the diagnosis of biliary tract carcinoma. 

In T cells, PKM2 is needed for T helper cells to achieve rapid differentiation, proliferation and biosynthesis [26]: upon TCR stimulation, PKM2 expression is increased [27,28]. Deletion of PKM2 inhibits the production of interferon (IFN)-γ [29], and prevention of dimerization of PKM2 severely impacts T cell activation [30]. In B cells, PKM2 expression and enzyme activity are increased during activation [31], proliferation [32] and antibody production [33]. However, the non-canonical functions of PKM2 remain to be investigated in these cells. Intracellular signalling between lymphocytes is crucial for the immune response: extracellular vesicles are a mechanism for this crosstalk. Yang et al. [34] demonstrated that T-cell–derived ceramide-filled extracellular vesicles regulate B cell IgG production via PKM2; PKM2-null vesicles inhibited B cell activation. In a different study [35], exosomes from B cells, isolated by their expression of MHC II, contained PKM2 that co-immunoprecipitated with MHC II complexes. The association of PKM2 to the ITAM motifs on the MHC II was postulated due to the association of PKM2 to the ITAM motifs of the Fc-ε RI receptor in mast cells: PKM2 is deactivated in mast cell degranulation via this interaction [36].

Periodontal diseases are chronic inflammatory diseases that share pathologies with other chronic inflammatory diseases. Periodontal diseases range from reversible gingivitis to irreversible periodontitis [37]. In the latter, destruction of the tooth-supporting tissues may eventually lead to tooth loss. Periodontitis is induced by the accumulation of a dysbiotic subgingival biofilm and exacerbated and sustained by the immune response [38]. Periodontal disease, in vivo, has been explored by the analysis of gingival crevicular fluid (GCF), which is sampled adjacent to affected teeth, and also by the analysis of saliva, which gives a more general view of the biological fluid composition of the whole mouth [39]. Proteomics, both mass spectrometry and gel-based, has been used to explore both these fluids. PKM2 has been identified in GCF of healthy people [40] and from donors with periodontitis [41,42,43,44] and gingivitis [45] in GCF and saliva. However, sometimes there is inconsistency as to whether there is more PKM2 in the healthy or inflammatory state, and it has rarely reached an increase over 2-fold. Davis et al. [44] found that PKM2 was increased in GCF from dogs naturally progressing from gingivitis to the early stages of periodontitis, whereas Bostanci et al. [41] found PKM2 in saliva to be higher in periodontitis donors. In recent work, Grant et al. (unpublished manuscript in preparation) demonstrated that PKM2 increased modestly in gingivitis but decreased in periodontitis in saliva. These data suggest that there is tight control on PKM2 in extracellular spaces within the mouth and that there is a biphasic release related to the severity or chronicity of the inflammation. However, there is no doubt about the contribution of the immune system to periodontal disease. Thorbert-Mros et al. [46] visualised the composition of gingival biopsies from chronic gingivitis and chronic periodontitis patients by immunohistochemistry for plasma cells, macrophages, neutrophils, B cells and T cells; demonstrating an expansion of all cell types and an overabundance of plasma cells in periodontitis. Li et al. [47], using a bioinformatic approach, classified transcriptomic data by CIBERSORT analysis into different immune cell subsets. They concluded that the transcript signatures identified more plasma cells, naive B cells and neutrophils in periodontitis versus healthy gingival biopsies. Neutrophils release PKM2 during the initial stages of inflammation, and this contributes to wound repair [14]: this may give rise to increases in gingivitis but not in periodontitis. However, this is speculation at this point. Neutrophils will also migrate through the periodontal tissues into the oral cavity, where they can be isolated from oral rinses as oral neutrophils. The transcriptome of oral neutrophils has been explored by Lakschevitz et al. [48] demonstrating a shift to a pro-survival phenotype in periodontal disease and transcriptomics of peripheral blood neutrophils stimulated with periodontopathogen *Fusobacterium nucleatum* has shown increases in hexokinase [49] but not in PKM2. Changes in activity may alter without changes in expression, however there are no reports yet on this. Thus, overall, there is a lack of information of the role of PKM2 in periodontal disease and there is a need to explore further the contribution of PKM2 to periodontal health and disease.

## Figures and Tables

**Figure 1:**
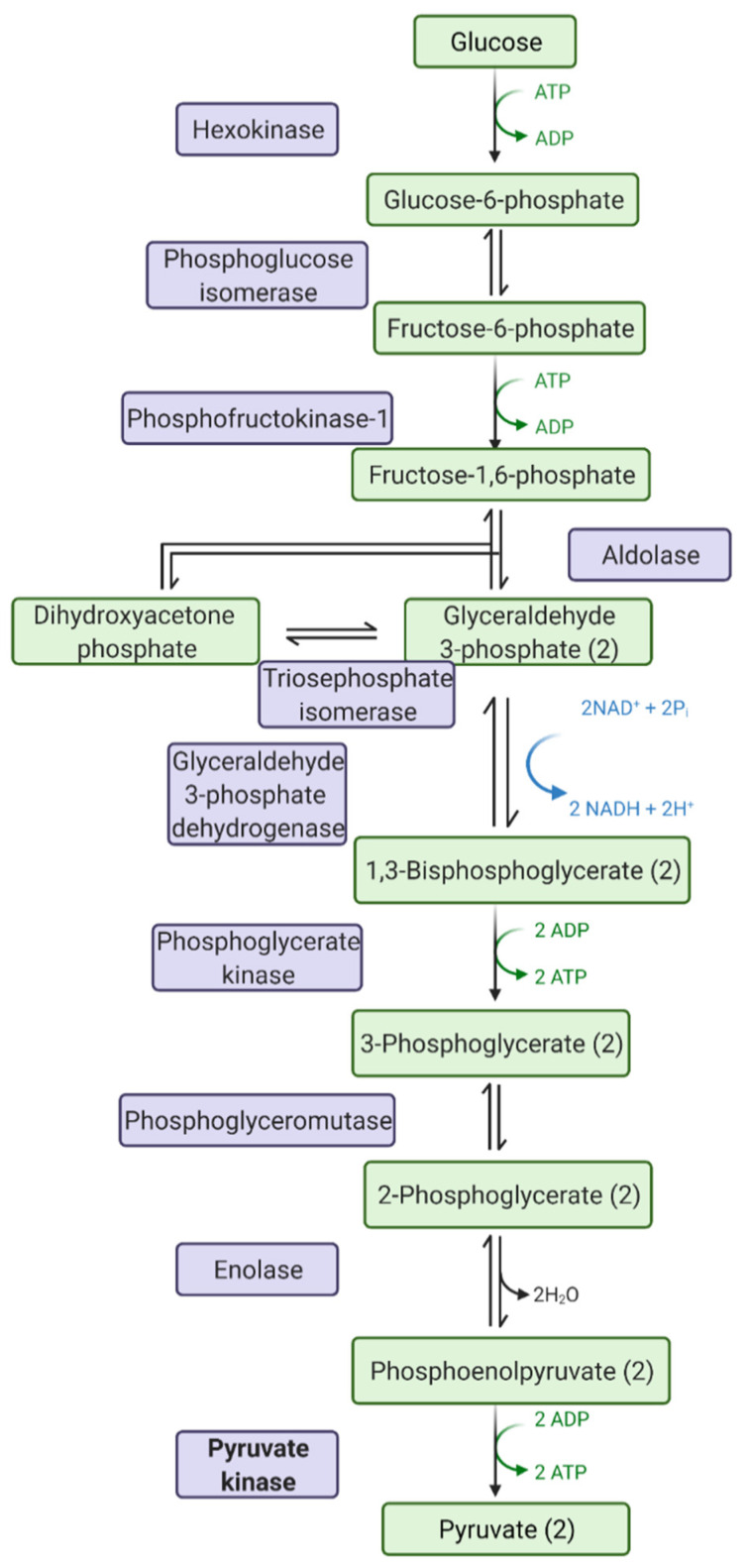
Steps in glycolysis: chemicals highlighted in green, and enzymes highlighted in purple. Pyruvate kinase is the last rate-limiting step in glycolysis. Image drawn with Biorender.com (accessed on 1 May 2021).
